# Supplementary material for: Numerical modelling of the 1 July 2022 flooding event, Southwest Huvadhoo Atoll, Maldives: Implications for the future
Source: Camb Prism Coast Futur. 2025 Oct 13;3:e23. doi: 10.1017/cft.2025.10013 (PMC12645334; doi:10.1017/cft.2025.10013)
Supplement: Masselink et al. supplementary material [file S2754720525100139sup001.docx]

Supporting information for

**Numerical modelling of the 1 July 2022 flooding event, Southwest Huvadhoo Atoll, Maldives: Implications for the future**

**Gerd Masselink^1^, Tim Poate^1^, Floor Roelvink^1,2^, Tim Scott^1^ and Robert McCall^2^**

^1^Coastal Processes Research Group, University of Plymouth, Plymouth, UK

^2^Deltares, Delft, Netherlands

**Contents of this file**

Figures S1 to S6

Tables S1 to S3

| 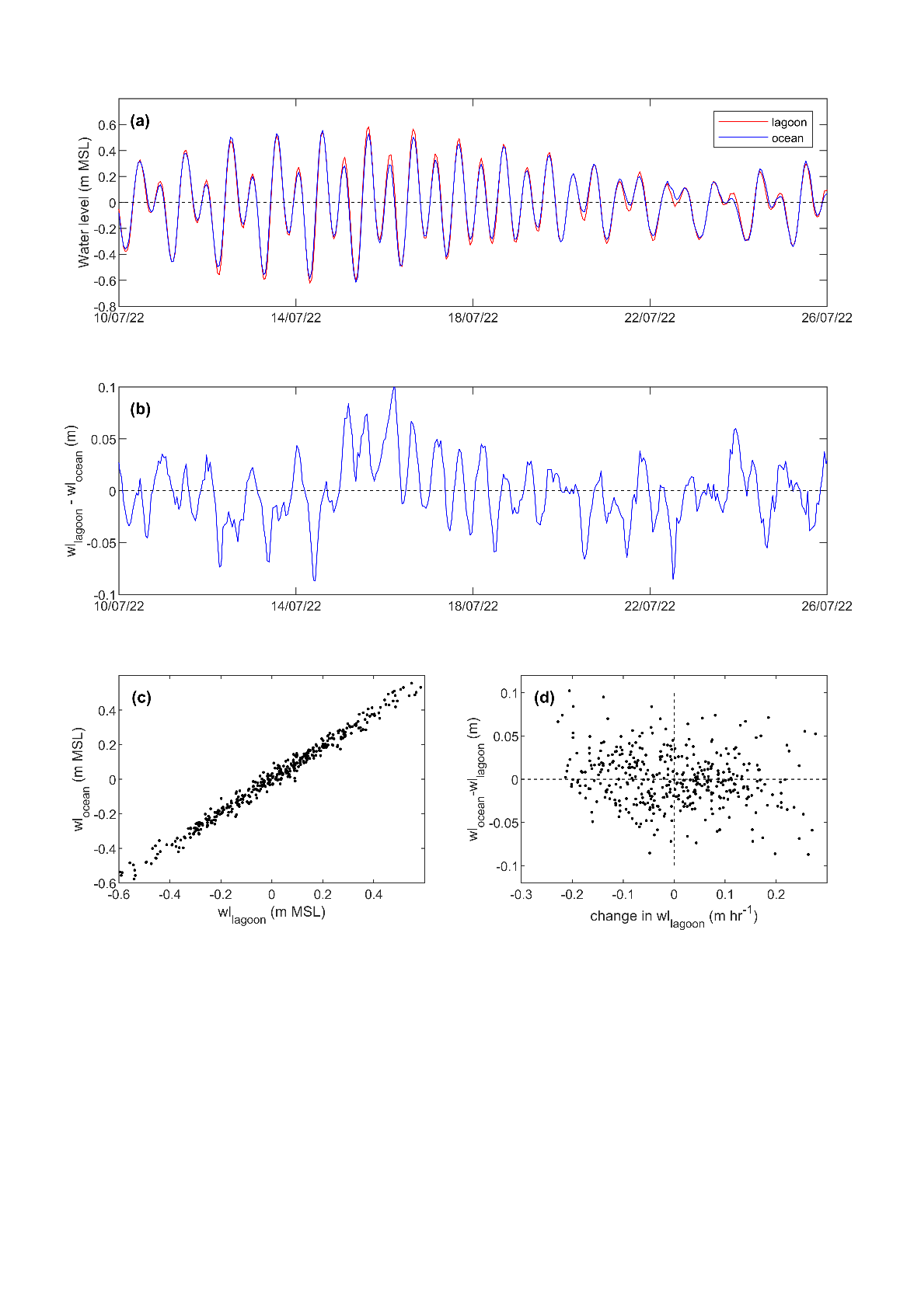 |
| --- |
| **Figure S1** – Time series of (a) detrended water level *wl* recorded in the harbour and in the ocean over a neap-to-neap tidal cycle; and (b) difference between the time series over the same period. Scatter plots of (c) ocean water level and lagoon water level; and (d) difference in water level between ocean and lagoon and the rate of lagoon water level change. Data points represent hourly-averaged data. |

| 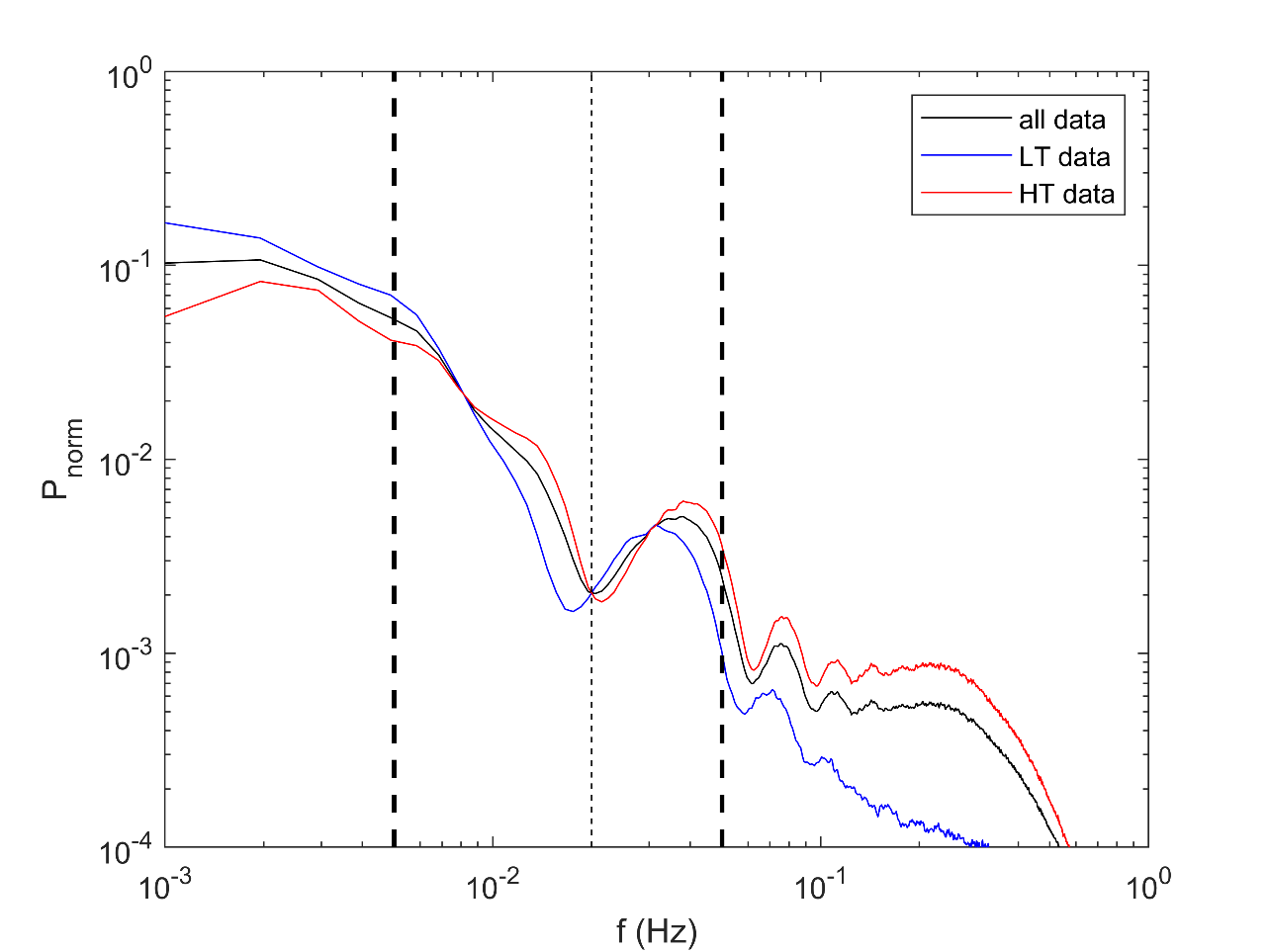 |
| --- |
| **Figure S2** – Mean normalised wave spectrum *P_norm_* computed from 1-hr pressure sensor data segments collected by the most inshore pressure sensor M1 for the period 18–31 July 2022, taking into account all data segments (black line), data collected for offshore water level > 0 m (HT data; red line) and < 0 m (LT data; blue line). Thick vertical dashed lines indicate the frequency cut-off used for very low frequency (VLF; *f* < 0.005 Hz) and infragravity (IG; *f* = 0.005–0.05 Hz) energy. The thin dashed line represents the frequency used for the low-pass filter for the wave group analysis in Figure **S3** (0.02 Hz). |

| 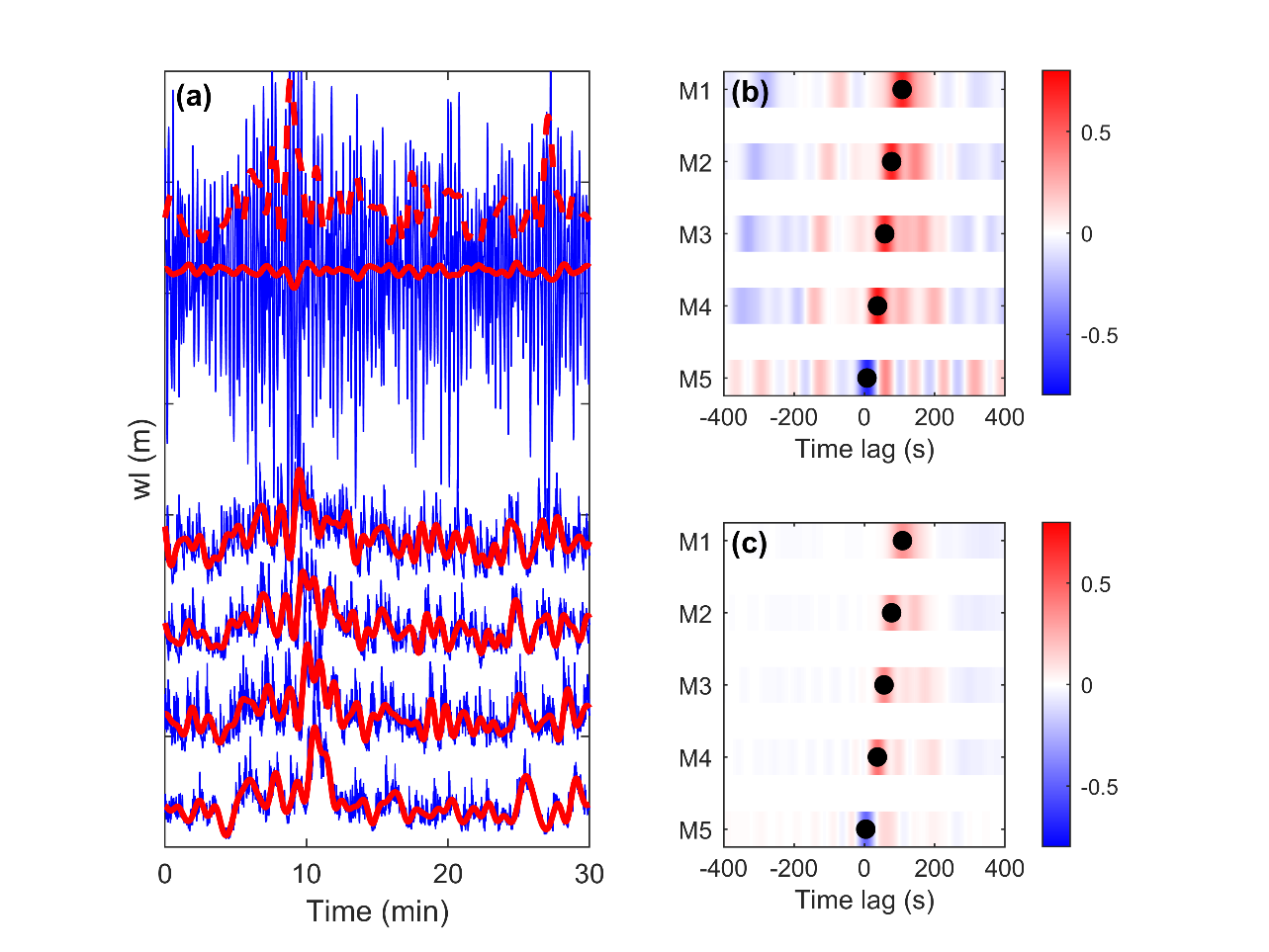 |
| --- |
| **Figure S3** – (a) Half-hour segment of 1-hr detrended time series of water level *wl* derived from (from top to bottom) the offshore pressure sensor M5 and those deployed at M4, M3, M2 and M1 (blue lines), with associated lowpass-filtered time series (red lines) obtained using a simple Fourier lowpass filter with cut-off frequency of 0.02 Hz, corresponding to the spectral valley between the IG and VLF wave frequency. The time series have been vertically offset by 0.5 m to aid visual comparison. The data were collected 20:30-21:30 on 19/07/22, with *H_s.25m_* = 1.50 m, *T_p_* = 17.0 s and *wl* = 0.04 m MSL. (b) Cross correlations for time series shown in (a), between lowpass-filtered groupiness envelope *GF* at offshore station (M5) with the  lowpass filtered water level time series at each instrument location on the reef platform (M4–M1). The black circles denote the peak in the cross-correlations. (c) The mean of the individual cross-correlations for all hourly bursts over the period 18–31 July 2022 for which the offshore water level *wl* > 0 m (HT data). |

| 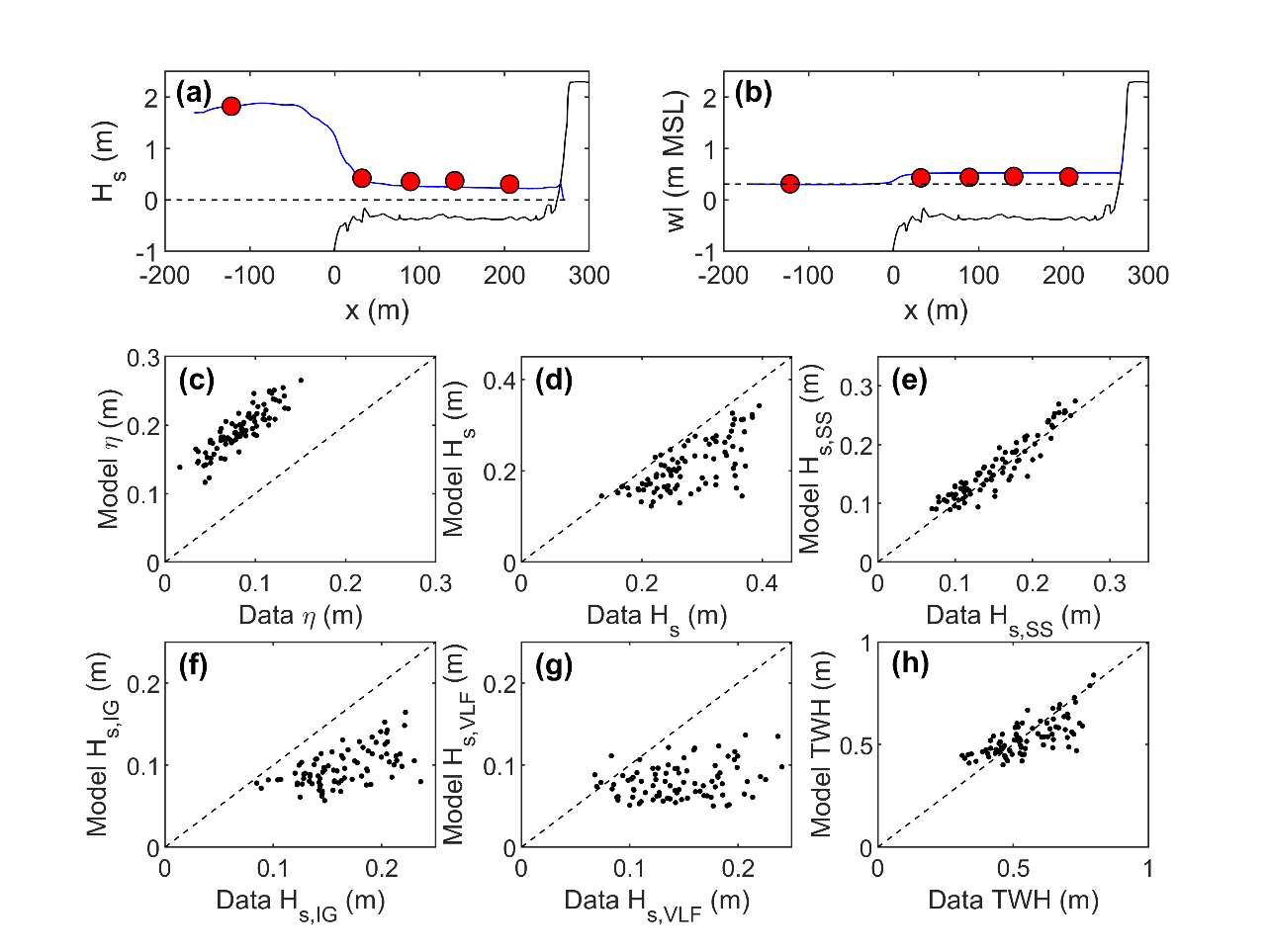 |
| --- |
| **Figure S4** – Results of the model calibration with measured topographic profile, *cf* of 0.3 across the spur-and-groove region (*h* = 0–10 m) and *cf* = 0.01 in the deeper water, across the reef platform and on the beach. (a) and (b) show comparison between model (line) and measured (red circles) *H_s_* and wave setup *η*, respectively, for one of the most energetic wave conditions of the calibration data set. (e), (f), (g) and (h) are scatter plots of measured data compared to modelled predictions of wave setup *η*, SS wave height *H_s,SS_*, IG wave height *H_s,IG_*, VLF wave height *H_s,VLF_* and total water height TWH (= *η* + *H_s,SS_* + *H_s,IG_* + *H_s,VLF_*), respectively. The measured wave conditions represent the inner-most PT (M1). |

| 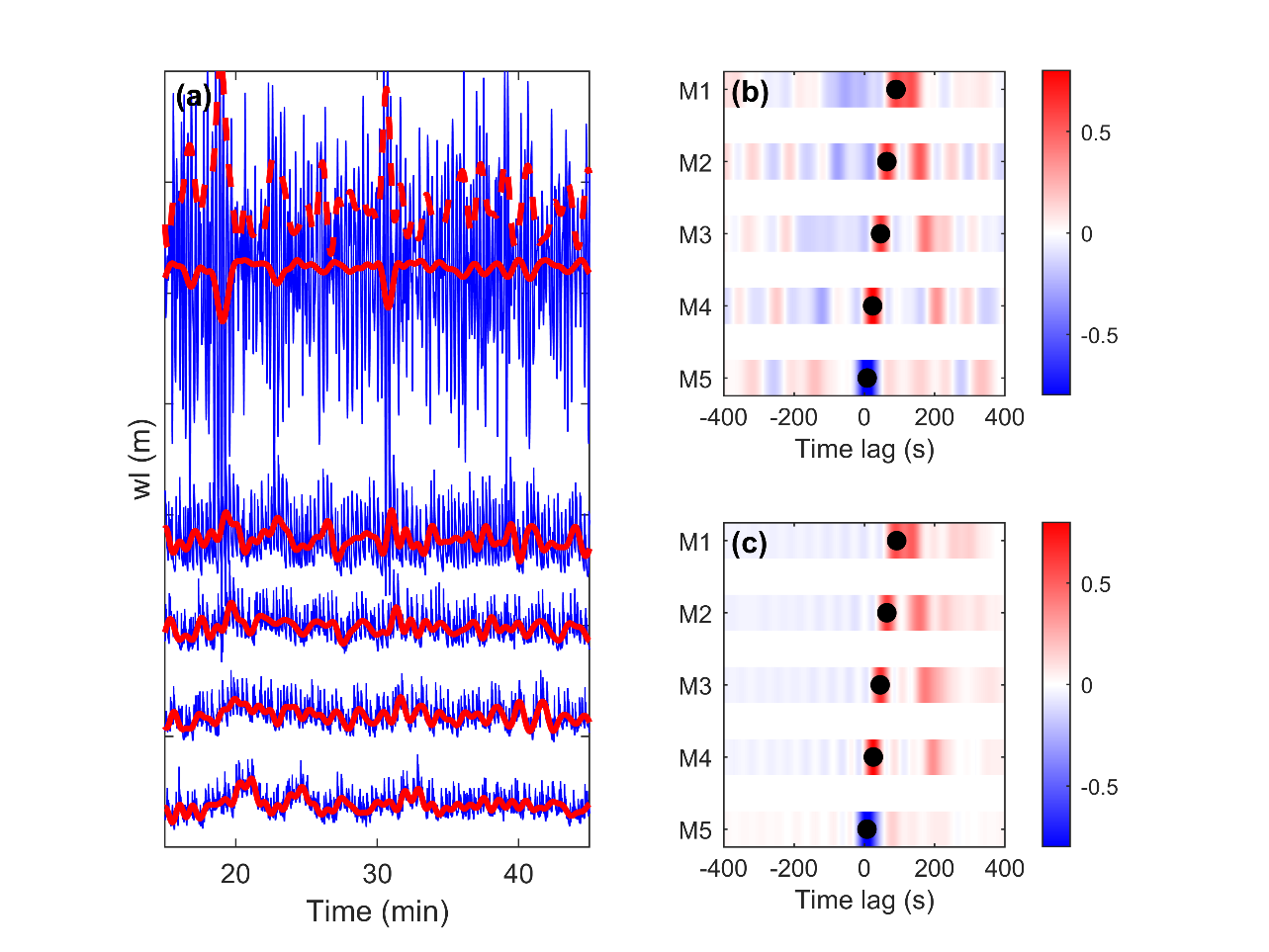 |
| --- |
| **Figure S5** – Same cross-correlation analysis between offshore wave groups and lowpass-filtered water level *wl* time series as presented in Figure **S3** (see caption of that figure), but based on modelled data. Panels (a) and (b) represent the same data segment as presented in Figure **S3** (20:30-21:30 on 19/07/22, with *H_s,25m_* = 1.50 m, *T_p_* = 17.0 and *wl* = 0.04 m MSL), while panel (c) is based on the full validation data set. |

| 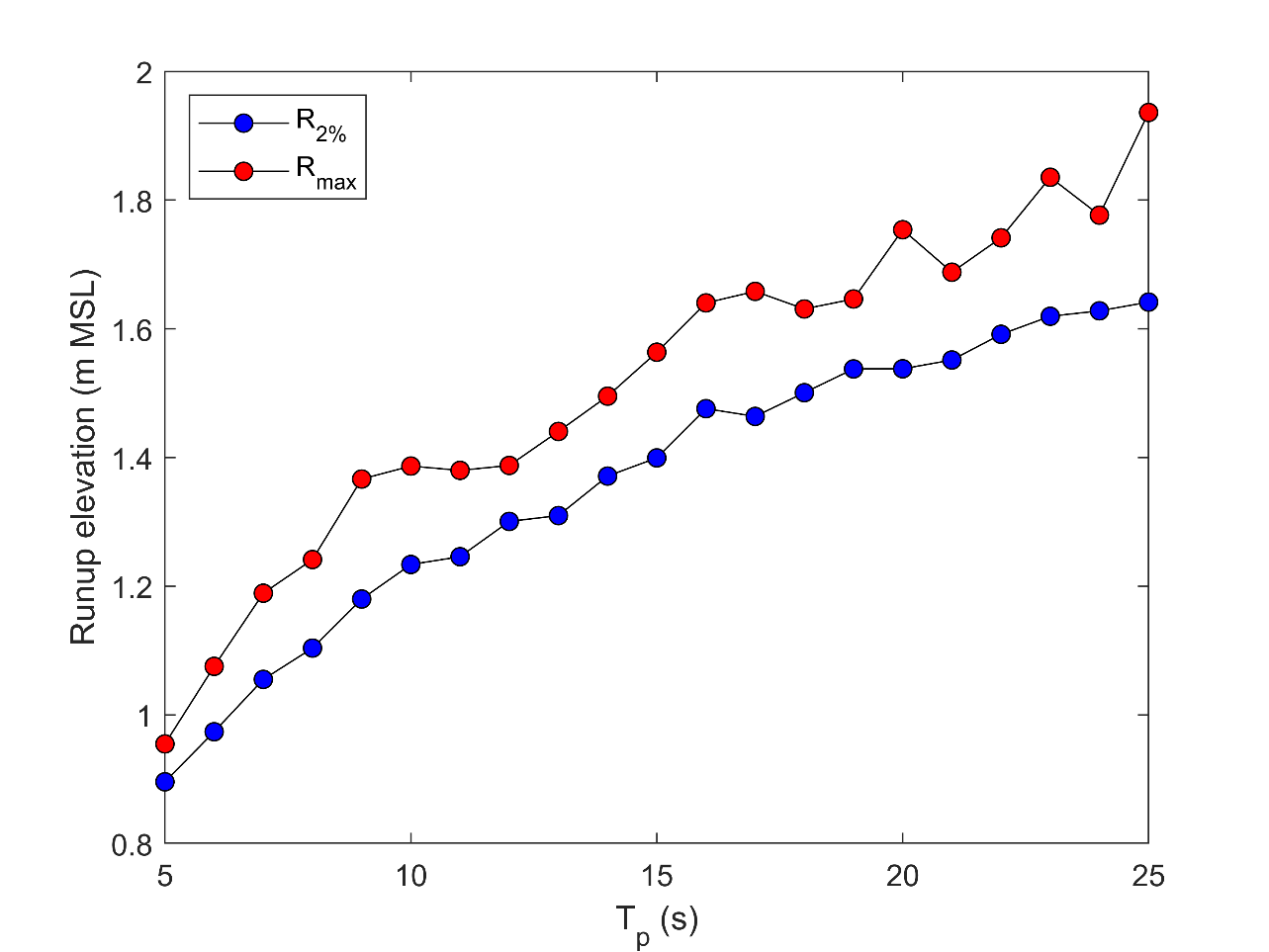 |
| --- |
| **Figure S6** – The modelled 2% exceedance runup elevation *R*_2%_ and maximum runup elevation *R_max_* as a function of peak wave period *T_p_* for constant water level *wl* = 0.53 m MSL and deep water wave height *H_s,o_* = 3.0 m using the same model parameters as those used for deriving Fig. **14**. The variability in the curves is due to the stochastic wave forcing in the numerical model, and is especially pronounced for *R_max_*. |

**Table S1** – Summary of average hydrodynamic conditions on the reef platform for the period 18–25 July 2022 (offshore wave height ranging from 1.1 m to 2.1 m), and for offshore water level > 0 m to avoid small water depths and/or standing water on the reef platform. *η* = wave setup, *H_s_* is total wave height, *H_s,SS_* is SS wave height (frequency > 0.05 Hz) and *H_s,IGVLF_* = IG and VLF wave height (frequency < 0.05 Hz). Value in parentheses represent s.d. associated with the mean.

|  | ***η* (m)** | ***H_s_* (m)** | ***H_s,SS_* (m)** | ***H_s,IGVLF_* (m)** |
| --- | --- | --- | --- | --- |
| **M1** | 0.11 (0.06) | 0.27 (0.06) | 0.14 (0.04) | 0.23 (0.06) |
| **M2** | 0.11 (0.06) | 0.31 (0.06) | 0.19 (0.05) | 0.23 (0.05) |
| **M3** | 0.10 (0.06) | 0.29 (0.05) | 0.17 (0.04) | 0.23 (0.05) |
| **M4** | 0.09 (0.06) | 0.34 (0.06) | 0.22 (0.05) | 0.25 (0.06) |
| **E** | 0.11 (0.07) | 0.33 (0.06) | 0.15 (0.04) | 0.28 (0.06) |
| **W** | 0.09 (0.07) | 0.27 (0.06) | 0.11 (0.04) | 0.24 (0.06) |

**Table S2** – Model performance for different values of bed roughness *cf* for the calibration data set. RMSE = root mean square error and *r* = correlation coefficient. Positive (negative) values for the bias in green (red) indicate that XBeach overpredicts (underpredicts). Only the wave conditions measured at inner-most PT (M1) position have been used in the model calibration.

|  | **RMSE (m)** | **Bias (m)** | ***r* (-)** |
| --- | --- | --- | --- |
| ***cf* = 0.01 everywhere (#26)** | | | |
| Setup | 0.16 | 0.16 | 0.93 |
| *H_s_* | 0.09 | 0.08 | 0.80 |
| *H_s,SS_* | 0.02 | 0.02 | 0.93 |
| *H_s,IGVLF_* | 0.10 | 0.09 | 0.66 |
| TWH | 0.30 | 0.30 | 0.85 |
| ***cf* = 0.1 on forereef; 0.01 elsewhere (#32)** | | | |
| Setup | 0.14 | 0.14 | 0.93 |
| *H_s_* | 0.05 | -0.03 | 0.76 |
| *H_s,SS_* | 0.02 | 0.01 | 0.94 |
| *H_s,IGVLF_* | 0.06 | -0.04 | 0.56 |
| TWH | 0.12 | 0.11 | 0.83 |
| ***cf* = 0.15 on forereef; 0.01 elsewhere (#25)** | | | |
| Setup | 0.11 | 0.11 | 0.89 |
| *H_s_* | 0.08 | -0.07 | 0.69 |
| *H_s,SS_* | 0.02 | 0.01 | 0.93 |
| *H_s,IGVLF_* | 0.10 | -0.09 | 0.40 |
| TWH | 0.08 | 0.00 | 0.74 |
| ***cf* = 0.2 on forereef; 0.01 elsewhere (#28)** | | | |
| Setup | 0.1 | 0.10 | 0.90 |
| *H_s_* | 0.09 | -0.08 | 0.70 |
| *H_s,SS_* | 0.02 | 0.00 | 0.92 |
| *H_s,IGVLF_* | 0.11 | -0.10 | 0.52 |
| TWH | 0.08 | -0.03 | 0.78 |
| ***cf* = 0.3 on forereef; 0.01 elsewhere (#27)** | | | |
| Setup | 0.07 | 0.07 | 0.90 |
| *H_s_* | 0.11 | -0.10 | 0.71 |
| *H_s,SS_* | 0.02 | 0.01 | 0.93 |
| *H_s,IGVLF_* | 0.14 | -0.14 | 0.55 |
| TWH | 0.14 | -0.12 | 0.79 |

**Table S3** – Results of XBeach model validation. RMSE = root mean square error and *r* = correlation coefficient. Positive (negative) values for the bias in green (red) indicate that XBeach overpredicts (underpredicts). Only the wave conditions measured at inner-most PT (M1) position have been used in the model validation.

|  | **RMSE (m)** | **Bias (m)** | ***r* (-)** |
| --- | --- | --- | --- |
| ***cf* = 0.15 on forereef; 0.01 elsewhere (#33)** | | | |
| Setup | 0.12 | 0.12 | 0.95 |
| *H_s_* | 0.07 | -0.06 | 0.79 |
| *H_s,SS_* | 0.03 | 0.01 | 0.83 |
| *H_s,IGVLF_* | 0.09 | -0.08 | 0.76 |
| TWH | 0.07 | 0.06 | 0.93 |
